# Suspected Post-ictal Psychosis in Temporal Lobe Epilepsy Secondary to Human Herpesvirus 6 Encephalitis

**DOI:** 10.7759/cureus.18535

**Published:** 2021-10-06

**Authors:** Jack Stover, Madhav Patel, Kathleen Carter, Keshav Patel

**Affiliations:** 1 Internal Medicine, Western Michigan University Homer Stryker School of Medicine, Kalamazoo, USA; 2 Neurology, Georgetown University M.D. School of Medicine, Washington, DC, USA; 3 Psychiatry, Western Michigan University Homer Stryker School of Medicine, Kalamazoo, USA

**Keywords:** hhv-6 encephalitis, temporal lobe epilepsy, post-ictal psychosis, psychosis, epilepsy, encephalitis

## Abstract

Human herpesvirus 6 (HHV-6) may lead to temporal lobe epilepsy (TLE). Psychotic syndrome frequency in the setting of TLE is 7% to 11%. We report a case of post-ictal psychosis (PIP) secondary to TLE in the setting of HHV-6 encephalitis. A 58-year-old male presented with a two-day history of severe confusion, personality changes, and new-onset psychosis. Lumbar puncture was positive for HHV-6. Electroencephalogram (EEG) revealed left temporal sharp waves during drowsiness and sleep, suggestive of focal epileptiform discharges without clinical seizures. Valproate and olanzapine were employed for epilepsy and agitation. Psychosis and confusion resolved with subsequent discharge. Out of the other diagnoses, PIP in the setting of TLE secondary to HHV-6 given the clinical response to acyclovir. While HHV-6 encephalitis may cause TLE, this patient did not have a history of seizures and EEG did not capture active seizures. It is unclear if the sharp waves were incidental or indicative of TLE. Additionally, PIP is seen more commonly with left-sided EEG changes. Low-dose olanzapine was efficacious in resolving symptoms, which is typical in PIP. Both HHV-6 encephalitis and TLE have the potential to cause memory impairments and personality changes, which were seen in this patient. Patients with both TLE and PIP are less likely to exhibit focal ictal discharges than those with only TLE, which may explain the absence of active seizure activity on EEG.

## Introduction

Human herpesvirus 6 (HHV-6) is a common virus, with approximately 90% of the world’s population exposed within the first three years of life [1]. While most patients are asymptomatic, HHV-6 can cause roseola and is associated with numerous central nervous system (CNS) complications. As is typical of many herpesviruses, HHV-6 can establish lifelong infection. HHV-6 has a particular propensity for establishing latent CNS infection due to its ability to replicate in multiple types of nervous tissue [2]. Reactivation events typically occur in immunocompromised individuals but can also infrequently occur in those without underlying immunological deficits. One such event is temporal lobe epilepsy (TLE) [3-5]. In addition to the concern of primary seizure complications, TLE is associated with dangerous sequelae such as post-ictal psychosis (PIP). The prevalence of psychotic syndromes, such as PIP, in the setting of TLE is between 7% and 11% [6]. We report the case of a 58-year-old immunocompetent male that presented with new-onset psychosis in the setting of TLE secondary to HHV-6 encephalitis.

## Case presentation

A 58-year-old male presented with a one-week history of confusion, personality changes, and new-onset psychosis, with severe exacerbation of these symptoms in the last two days. He reported homicidal ideations and exhibited bizarre delusions of being an alien. He displayed impulsivity, irritability, tangentiality, and flight of ideas. The patient was oriented to person, date, and knew he was in a hospital but did not know the city it was located. According to his wife, this would be information he would otherwise know. The patient had no diurnal fluctuations in mental status. Due to the patient’s altered mental status, his wife provided the supplemental history. The patient also reported urinary frequency, which was attributed to glucosuria secondary to reported recent noncompliance with the home diabetes medication regimen. The patient had an unintentional weight loss of 22.68 kg over the past six-months, which was attributed to significantly increased personal stress. The review of systems was otherwise negative. Past medical history was significant for type 2 diabetes mellitus and major depressive disorder. The patient started escitalopram two months prior and admitted to occasional marijuana use but denied any changes to frequency or amount. Of note, he was hospitalized one week prior for mild confusion, reduced consciousness, aphasia, nausea, and headache.

The physical exam was unremarkable. Head, abdominal, pelvic computed tomography, and chest x-ray were unremarkable. Complete blood count, complete metabolic panel, serum thyroid-stimulating hormone (TSH), serum magnesium, and serum folate were all unremarkable. Urinalysis unremarkable save the presence of +3 glucosuria. Gram stain of cerebrospinal fluid (CSF) and blood was negative. The urine drug screen was positive only for cannabinoids. Serum glucose was 277 mg/dL with a hemoglobin A1C of 7.5. CSF findings included increased glucose and protein without any red blood cells or white blood cells. Lumbar puncture was polymerase chain reaction (PCR)-positive for HHV-6 and acyclovir was started. There was the resolution of symptoms over the next five days, and the patient was discharged with orders to finish the acyclovir regimen at home. The patient received no antipsychotic or antiepileptic medications during this hospitalization. 

Two days after discharge, the patient was brought to the emergency department by the police due to a domestic dispute which was precipitated by recurrence of his psychotic symptoms. Repeat comprehensive metabolic panel and complete blood count were both unremarkable. Repeat urine drug screen was again positive for cannabis. After consultation with infectious disease, it was determined that his current symptoms were unlikely to have an acute infectious etiology given his original response to antiviral treatment.

Magnetic resonance imaging of the brain with and without contrast was unremarkable. Electroencephalogram (EEG) revealed sharp left temporal waves during drowsiness and sleep, suggestive of focal epileptiform discharges without clinical seizures. This was consistent with TLE, especially in the context of his recent HHV-6 episode. Interestingly, the patient and his wife reported no history of seizures or sequelae thereof. 250 mg oral valproate at morning and midday with 750 mg dose at bedtime and 5 mg olanzapine oral at bedtime were started for epilepsy and agitation. Within one-week, psychosis and confusion resolved with subsequent discharge on 500 mg oral valproate twice a day and 5 mg oral olanzapine at bedtime with instructions to revisit these medications at outpatient neurology follow-up in two weeks. The patient was ultimately weaned off olanzapine within a year and continued on valproate indefinitely.

## Discussion

The patient’s response to acyclovir and positive HHV-6 CSF PCR during his initial hospitalization strongly suggested reactivation of HHV-6 as the underlying cause of his new-onset neurologic symptoms. Adult disease due to HHV-6 is almost always due to reactivation [7]. His EEG was highly suggestive of TLE, a common consequence of HHV-6 reactivation. Up to 70% of patients with TLE had increased HHV-6 DNA load in resected temporal lobe tissue compared to the general population [8]. While the patient reported no seizure history and an active seizure event was not recorded on EEG, sharp waves of the left temporal lobe during drowsiness and sleep consistent with TLE were seen [9]. Furthermore, these abnormalities persisted on follow-up outpatient EEGs.

The patient’s TLE was likely further complicated by PIP given the temporal relationship between his original symptom response to acyclovir and subsequent response to olanzapine, which is typical of PIP. TLE is associated with PIP and diagnostic criteria for PIP allows for its development up to a week after a seizure episode [10]. Additionally, PIP is seen more commonly with left-sided EEG changes [6]. Both HHV-6 encephalitis and TLE can cause memory impairments and personality changes, which were seen in this patient. Patients with both TLE and PIP are also less likely to exhibit focal ictal changes than those with only TLE, which may explain the absence of active seizure activity on EEG [10]. 

The patient’s recent 22.68 kg weight loss was an area of suspicion and curiosity. HHV-6 reactivation is far more common in immunocompromised patients which, when considered in the context of HHV-6 reactivation, raises the possibility of malignancy [11]. However, imaging and labs found no such evidence and weight loss was thus attributed to his recent high-stress state and malnutrition. Even so, there are prior cases reported of immunocompetent adults presenting with HHV-6 encephalitis [12]. 

Finally, cannabis-induced psychosis was considered as a possible etiology of his symptoms. This was ultimately considered less likely given his positive response to antivirals, positive HHV-6 CSF PCR, and EEG characteristic of TLE. Successful treatment with antipsychotic and antiepileptic medication during the patient’s repeat hospitalization suggests these psychotic symptoms are likely due to PIP caused by TLE from initial HHV-6 reactivation. Furthermore, the patient denied any changes in marijuana consumption and remained asymptomatic after the second discharge despite reporting no changes to marijuana use at follow-up appointments.

## Conclusions

This case emphasizes the importance of considering HHV-6 reactivation as a cause of new-onset neurologic disturbances even in immunocompetent patients. Providers should also consider EEG evaluation in patients found to have symptoms and CSF results consistent with HHV-6 reactivation for possible TLE, even in the absence of overt epileptic symptoms. However, negative EEG findings should not exclude the diagnosis of TLE, especially in the context of PIP. Finally, this case report encourages the consideration of PIP as a cause of psychosis in patients with a recent history of HHV-6 encephalitis and TLE.
